# Editorial: Natural products, medicinal foods and complementary and alternative medicine as cancer-preventive agents

**DOI:** 10.3389/fphar.2023.1232249

**Published:** 2023-07-06

**Authors:** Muhammad Zia-Ul-Haq, Romina Alina Marc, Muhammad Riaz

**Affiliations:** ^1^ ORIC (Office of Research Innovation and Commercialization), Lahore College for Women University, Lahore, Pakistan; ^2^ Department of Food Science, Faculty of Food Science and Technology, University of Agricultural Sciences and Veterinary Medicine Cluj-Napoca, Cluj-Napoca, Romania; ^3^ Department of Pharmacy, Shaheed Benazir Bhutto University, Sheringal, Pakistan

**Keywords:** natural products, medicinal foods, complementary, alternative medicine, cancer-preventive agents

Cancer is among the leading cause of death despite established treatment approaches, i.e., chemotherapy, radiotherapy and surgery, since none of the approaches are 100% effective. It is estimated that the causative agent of every sixth death globally is cancer (Arnold et al., 2022). Adverse outcomes are usually the major effects associated with treatment approaches, hence there is growing interest in exploring alternative strategies for cancer prevention. Natural products, medicinal foods, and complementary and alternative medicine (NP-MF-CAM) have gained attention as potential cancer-preventive agents. This Research Topic aims to showcase all relevant investigations about these approaches in reducing the risk of cancer and their integration into mainstream cancer prevention strategies.

A total of 54 manuscripts were submitted to this Research Topic, of which only six were accepted, (three review articles and three research articles). The three review articles are about the antiproliferative potentials of salvianolic acid B, astragaloside IV (AS-IV), and aconitine. These metabolites are isolated from medicinal plants that are well-known in traditional Chinese medicine (TCM) for their anti-tumor use. One review article described the anticancer potential of salvianolic acid B, isolated from Salvia miltiorrhiza Bunge (Xia et al.), which induces apoptosis in cancer cells by promoting ROS production and regulating energy metabolism, while another review highlighted the anticancer potential with the mechanism of AS-IV isolated from Radix Astragali, which has been neglected as an effective adjuvant drug for cancer treatment (Xia et al.; Guo and Wang). In the third review article, it was confirmed after a systematic review of preclinical studies that aconitine has a strong anti-tumor effect; however, further *in vivo* studies are required (Xiang et al.).

Of the research articles, one is about pre-clinical studies in which the use of traditional medicine in tumors was scientifically evaluated, and the botanically derived drugs from cultivated *Paris polyphylla* var. *yunnanensis* were explored for anticancer potential with significant results (Yan et al.). In a second research article, the TCM-based treatment, i.e., Huaier granules adjuvant therapy, was studied clinically, and the study showed that the overall survival rate in patients with hepatocellular carcinoma was improved (Shi et al.), which further strengthens the title of the Research Topic towards the management of cancer. A third article focuses on minimizing the adverse events associated with diagnosis procedures such as endoscopy or gastroscopy in diseases of the gastrointestinal tract early stage cancer (GIT) or early cancer. Propofol is given for anesthesia due to its quick onset prior to endoscopic procedures, but it may result in hypotension, bradycardia, and respiratory depression. The administration of esketamine significantly and dose-dependently reduced the dose of propofol required to accomplish procedures. Dose-related adverse events may be reduced with this combination (Feng et al.).

NP-MF-CAM offer a promising avenue for cancer prevention. The bioactive compounds present in these natural products, along with their potential synergistic effects, make them attractive candidates for further research and development. However, it is essential to access these approaches with a critical and evidence-based mindset, ensuring that rigorous scientific studies are conducted to validate their efficacy and safety. As we continue to explore innovative strategies for cancer prevention, the integration of NP-MF-CAM therapies into conventional healthcare systems can provide a more comprehensive approach to reduce the burden of cancer and improve overall public health.
